# Regional Lymph Node Involvement Among Patients With De Novo Metastatic Breast Cancer

**DOI:** 10.1001/jamanetworkopen.2020.18790

**Published:** 2020-10-09

**Authors:** Almir Bitencourt, Carolina Rossi Saccarelli, Elizabeth A. Morris, Jessica Flynn, Zhigang Zhang, Atif Khan, Erin Gillespie, Oren Cahlon, Boris Mueller, John J. Cuaron, Beryl McCormick, Simon N. Powell, George Plitas, Pedram Razavi, Katja Pinker, Christopher C. Riedl, Elizabeth J. Sutton, Lior Z. Braunstein

**Affiliations:** 1 Department of Radiology, Memorial Sloan Kettering Cancer Center, New York, New York; 2 Department of Imaging, A. C. Camargo Cancer Center, Sao Paulo, Brazil; 3 Department of Radiology, Hospital Sírio-Libanês, São Paulo, Brazil; 4 Department of Epidemiology and Biostatistics, Memorial Sloan Kettering Cancer Center, New York, New York; 5 Department of Radiation Oncology, Memorial Sloan Kettering Cancer Center, New York, New York; 6 Department of Surgery, Memorial Sloan Kettering Cancer Center, New York, New York; 7 Department of Medicine, Memorial Sloan Kettering Cancer Center, New York, New York

## Abstract

**Importance:**

Regional nodal irradiation (RNI) for node-positive breast cancer reduces distant metastases and improves survival, albeit with limited reduction in regional nodal recurrences. The mechanism by which RNI robustly reduces distant metastases while modestly influencing nodal recurrences (ie, the presumed target of RNI) remains unclear.

**Objective:**

To determine whether some distant metastases putatively arise from occult regional nodal disease and whether regional recurrences otherwise remain largely undetected until an advanced cancer presentation.

**Design, Setting, and Participants:**

This cohort study examined patients presenting with de novo stage IV breast cancer to the Memorial Sloan Kettering Cancer Center in New York, New York, from 2006 to 2018. Medical records were reviewed to ascertain clinicopathological parameters, including estrogen receptor status and survival. Pretreatment positron emission tomography–computed tomography (PET-CT) imaging was reviewed to ascertain the extent of regional nodal involvement at metastatic diagnosis using standard nodal assessment criteria. A subset underwent regional lymph node biopsy for diagnostic confirmation and served to validate the radiographic nodal assessment. Data analysis was performed from October 2019 to February 2020.

**Exposures:**

Untreated metastatic breast cancer.

**Main Outcome and Measures:**

The primary outcome was the likelihood of regional nodal involvement at the time of metastatic breast cancer presentation and was determined by reviewing pretreatment PET-CT imaging and lymph node biopsy findings.

**Results:**

Among 597 women (median [interquartile range] age, 53 [44–65] years) with untreated metastatic breast cancer, 512 (85.8%) exhibited regional lymph node involvement by PET-CT or nodal biopsy, 509 (85%) had involvement of axillary level I, 328 (55%) had involvement in axillary level II, 136 (23%) had involvement in axillary level III, 101 (17%) had involvement in the supraclavicular fossa, and 96 (16%) had involvement in the internal mammary chain. Lymph node involvement was more prevalent among estrogen receptor–negative tumors (92.4%) than estrogen receptor–positive tumors (83.6%). Nodal involvement at the time of metastatic diagnosis was not associated with overall survival.

**Conclusions and Relevance:**

These findings suggest that a majority of patients with de novo metastatic breast cancer harbor regional lymph node disease at presentation, consistent with the hypothesis that regional involvement may precede metastatic dissemination. This is in alignment with the findings of landmark trials suggesting that RNI reduces distant recurrences. It is possible that this distant effect of RNI may act via eradication of occult regional disease prior to systemic seeding. The challenges inherent in detecting isolated nodal disease (which is typically asymptomatic) may account for the more modest observed benefit of RNI on regional recurrences. Alternative explanations of nodal involvement that arises concurrently or after metastatic dissemination remain possible, but do not otherwise explain the association of RNI with distant recurrence.

## Key Points

**Question:**

Following resection of a localized breast cancer, why does adjuvant regional nodal irradiation (RNI) reduce distant recurrences to a greater extent than regional recurrences, despite only targeting the regional lymph nodes?

**Findings:**

In this cohort study of 597 patients with de novo metastatic breast cancer, 85.8% harbored regional disease at the time of metastatic presentation. The consistent detection of concurrent regional and metastatic disease, along with the reduction in distant recurrences observed following RNI, is consistent with the hypothesis that regional nodal disease may precede metastatic dissemination.

**Meaning:**

These findings suggest that RNI may reduce distant recurrences by sterilizing occult regional nodal disease prior to systemic seeding.

## Teaser

This cohort study examines whether and why regional node irradiation in patients with de novo metastatic breast cancer has different levels of effectiveness for preventing recurrences of cancer in regional nodes and distant mestastases.

## Introduction

It has long been observed that breast cancer outcomes can be improved with surgical and radiotherapeutic management of the regional lymph node basins.^1,2,3^ The relevant regional lymph nodes include the axilla (levels I-III), supraclavicular fossa, and the internal mammary nodal chain.^4^ Although surgical extirpation of these basins has been deescalated in recent years with the advent of sentinel lymph node biopsy,^5,6^ regional nodal irradiation (RNI) has played an increasing role in locoregional management. Indeed, significant technical advances in the multidisciplinary management of the axilla have reduced the morbidity of therapy while improving long-term disease control and survival.^7,8,9^

RNI for node-positive breast cancer reduces distant metastases (DM) and improves overall survival, even among patients with a low disease burden, although there have been reports of limited concomitant reduction in regional nodal recurrences.^10,11,12,13^ The mechanism by which RNI might reduce distant recurrences to a greater degree than regional recurrences remains unknown. Because posttreatment surveillance of regional nodal recurrences is limited, we hypothesized that some DM putatively arise from occult regional nodal disease (ie, the target of RNI), and that regional nodal recurrences may otherwise go undetected until an advanced cancer presentation. Here, we sought to investigate the likelihood of regional lymph node involvement among patients with de novo metastatic breast cancer, a population in whom the full natural history of breast cancer from localized to systemic disease has presumably taken place, permitting an anatomical accounting of whether the regional lymph nodes might represent an intermediary in the course of disease progression.

## Methods

This cohort study was approved by the Memorial Sloan Kettering Cancer Center’s institutional review board, which waived the requirement for informed patient consent because it was a retrospective data analysis. This study follows the Strengthening the Reporting of Observational Studies in Epidemiology (STROBE) reporting guideline. We identified 1314 patients with breast cancer who presented with de novo metastatic disease to our institution from January 2006 to September 2018. For this analysis, we excluded 521 patients with no analyzable pretreatment ^18^F-fluorodeoxyglucose (FDG) positron emission tomography–computed tomography (PET-CT) images, 119 with bilateral breast cancer, 55 who had undergone prior treatment, 16 with primary occult breast cancer, 5 male patients, and 3 patients with synchronous malignant neoplasms, yielding 597 analyzable patients reported here. Regional lymph node involvement was identified by pathologic evaluation (via nodal biopsy results, where available) and by central assessment of PET-CT for all patients.

PET-CT images and reports were reviewed by a single dedicated breast radiologist (A.B.) who assessed both morphological and metabolic features of regional lymph nodes. The reader was not blinded to the study objective, but was blinded to biopsy results among the subset of patients where they were available. Nodal morphological profile was classified as normal, mildly suspicious (cortical thickening), or abnormal (round shape, absent hilum or enlarged size). FDG uptake was classified as no abnormal uptake, mild uptake (maximum standardized uptake value <2.0), or moderate to high uptake (maximum standardized uptake value ≥2.0). Nodal status was then stratified according to the likelihood of true metastatic involvement using a standardized 5-category lexicon^14^: (1) unlikely (<10% probability), when there were no morphological changes or abnormal FDG uptake; (2) less likely (approximately 25% probability), when there were suspicious morphological features or mild FDG uptake; (3) possible (approximately 50% probability), when there were suspicious morphological features and mild FDG uptake; (4) suspicious (approximately 75% probability), when there were abnormal morphological features or moderate to high FDG uptake; and (5) consistent with (>90% probability), when there were abnormal morphological features and moderate to high FDG uptake (eFigure 1 in the Supplement). For the purposes of this analysis, patients were considered to have nodal involvement only if FDG PET-CT findings were suspicious or consistent with axillary lymph node metastasis (ie, >75% or >90% confidence, respectively); any assessments with lower confidence were considered not to have lymph node involvement. Positive lymph nodes were classified according to their location in axillary levels I, II, and III; the supraclavicular fossa; or the internal mammary chain (eFigure 2 in the Supplement).

Where available, the determination of axillary nodal involvement relied on pathological evaluation via percutaneous biopsy (fine needle aspiration or core needle biopsy) or surgical procedure (sentinel node biopsy or axillary dissection). Pathological results were considered for analysis only if they were centrally reviewed at our institution. Breast cancer subtypes were defined as luminal (estrogen receptor [ER] and/or progesterone receptor [PR] positive and *ERBB2* [formerly *HER2*] negative), *ERBB2* positive (ER positive or negative, PR positive or negative, and *ERBB2* positive), or triple negative (ER, PR, and *ERBB2* negative).

### Statistical Analysis

Statistical tests were all 2-sided, and the level of significance was set at *P* <. 05. The Fisher exact test was used to compare estrogen receptor status and subtype between patients who were node positive and patients who were node negative, and the Wilcoxon rank-sum test was used to compare the ages of patients who were node positive with patients who were node negative. The Kaplan-Meier method was used to estimate overall survival, which was ascertained from the medical record, and the log-rank test was used to compare survival outcomes by nodal involvement. Statistical analyses were conducted using R statistical software version 3.6.0 (The R Project for Statistical Computing) from October 2019 to February 2020.

## Results

**Figure 1.**
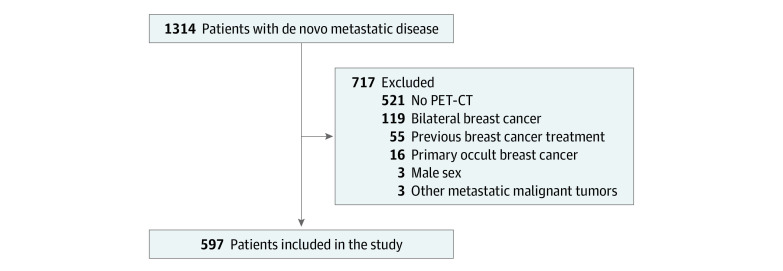
Patients With De Novo Metastatic Breast Cancer Who Were Exlcuded From the Study PET-CT indicates positron emission tomography–computed tomography.

Of 1314 patients presenting to our institution with de novo metastatic breast cancer, we excluded 717 as outlined in Figure 1, leaving 597 analyzable women (median [interquartile range] age, 53 [44–65] years) with PET-CT images that could be adequately evaluated. Of these 597 patients in the study, 525 (87.9%) had invasive ductal carcinoma. The luminal-like subtype was the most common (287 patients [56.9%]), followed by the *ERBB2-*positive subtype (154 patients [30.6%]) and the triple-negative subtype (63 patients [12.5%]).

Among this unselected cohort of patients with de novo metastatic breast cancer, PET-CT images showed evidence of regional lymph node involvement in 500 patients (83.8%), including 133 patients (22.3%) with radiographic imaging classified as suspicious and 367 patients (61.5%) with radiographic imaging classified as consistent with, as described in the Methods. The remaining 97 patients (15.2%) were classified as radiographically negative on PET-CT images (of these, 12 exhibited pathologic evidence of nodal involvement on biopsy: 9 of these had possible radiographic involvement on PET-CT, 2 had less likely involvement, and 1 had unlikely involvement). Thus, on final assessment by imaging and biopsy evidence, 512 of 597 patients with de novo metastatic breast cancer (85.8%; 95% CI, 82.7%–88.5%) exhibited regional nodal involvement.

Analysis of factors associated with nodal involvement revealed that biologic subtype and ER status were associated with final nodal status. Lymph node involvement was more prevalent among ER–negative tumors (92.4%) than ER–positive tumors (83.6%) (Table).

**Table.** Association Between Clinicopathologic Characteristics and Lymph Node Involvement

Characteristics	Participants, No. (%)		*P* value
	Negative lymph node involvement (n = 85)	Positive lymph node involvement (n = 512)	
Age, median (interquartile range), y	53 (44-65)	53 (44-64)	.70
Estrogen receptor^a^			
Negative	11 (7.6)	134 (92.4)	.009
Positive	71 (16.4)	363 (83.6)	
Subtype^b^			
Luminal	50 (17.4)	237 (82.6)	.04
*ERBB2 (formerly HER2)*	16 (10.4)	138 (89.6)	
Triple-negative	5 (7.9)	58 (92.1)	

^a^ Data were missing for 18 cases.

^b^ Data were missing for 93 cases.

**Figure 2.**
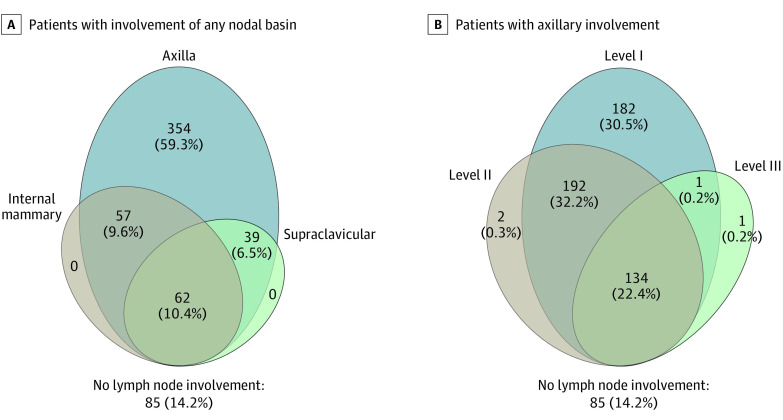
Types of Nodal Involvement for Patients With De Novo Metastatic Breast Cancer A, For patients with involvement of any nodal basin, various combinations of axilla, internal mammary, and supraclavicular involvement are shown. B, For patients with axillary involvement, various combinations of level I, level II, and level III involvement are shown.

Among those with nodal involvement, axillary level I was most frequently involved (509 patients [85%]), followed by axillary level II (328 patients [55%]), axillary level III (136 patients [23%]), the supraclavicular fossa (101 patients [17%]), and the internal mammary chain (96 patients [16%]). Of patients with nodal disease, 158 (26.5%) had involvement seen in more than 1 nodal basin, with the various combinations illustrated in Figure 2A. No patients had isolated supraclavicular or internal mammary disease without axillary involvement. Similarly, among those with axillary involvement, less than 1% exhibited isolated level II or III disease without involvement of level I (Figure 2B).

**Figure 3.**
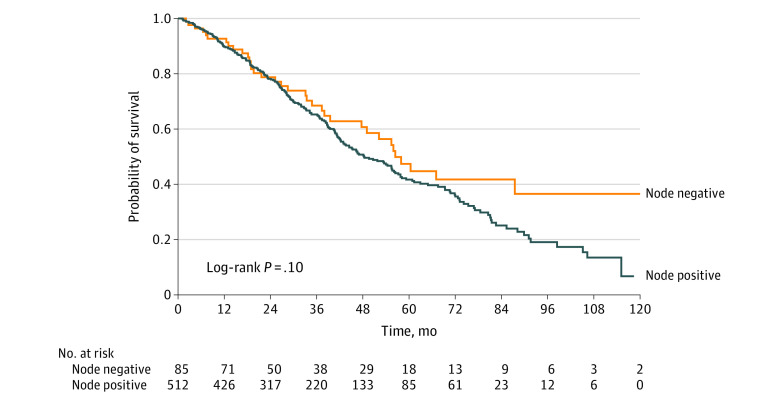
Kaplan-Meier Estimates of Survival for Patients With De Novo Metastatic Breast Cancer by Nodal Involvement

Overall survival at the 5-year mark was 42.4% (95% CI, 37.6%–47.9%) among the entire cohort (41.7% [95% CI, 36.5%-47.7%] among those with nodal disease, 47.4% [95% CI, 35.7%-62.9%] among those without). Survival analysis revealed that the presence or absence of concurrent regional disease at the time of metastatic presentation was not associated with overall survival (Figure 3).

## Discussion

Our findings illustrate that among an unselected and previously untreated cohort of patients with de novo metastatic breast cancer, most presented with evidence of regional nodal disease concurrent with the identification of DM. This observation lends support to the possibility that metastatic seeding may arise from uncontrolled nodal disease, and that nodal radiotherapy among patients with early-stage disease may otherwise mitigate distant spread by sterilizing occult regional disease that has yet to disseminate. With regard to anatomic delineation, axillary level I accounted for most of the observed nodal involvement, with only a few patients exhibiting isolated regional nodal disease in other basins.

Regional lymph node management has been a vigorous focus of breast cancer research for the better part of the past century. Beginning with Halstedian efforts to use increasingly aggressive nodal resection (eg, the radical and extended radical mastectomy), investigators have studied how best to balance iatrogenic toxicity with locoregional therapy. The NSABP B-04 trial^15^ provided early evidence that axillary dissection could be omitted in select populations and that radiation might play a role in postmastectomy nodal management. The ACOSOG Z0011^6^ and AMAROS^5^ trials thereafter demonstrated that even among patients with positive sentinel nodes, axillary dissection could be avoided in those receiving adjuvant radiation to the breast or regional nodes, respectively. More recently, a series of landmark prospective randomized studies^11,12^ further illustrated the role of RNI among those with a limited axillary disease burden.

Both the MA.20^11^ and EORTC 22922^12^ trials randomized several thousand patients who were node-positive or high-risk node-negative to the receipt of RNI targeting the axilla, supraclavicular fossa, and internal mammary chain. Whereas both trials showed a modest improvement in 10-year regional control following RNI (2% regional recurrence benefit in MA.20 and 1.5% benefit in EORTC 22922), the reduction in distant recurrences was paradoxically more substantial (3.6% in MA.20 and 3.7% in EORTC 22922). This reduction in DM translated to the now oft-quoted disease-free survival benefit of RNI (10-year benefit of 5% on MA.20 and 3% on EORTC 22922).^11,12^ Moreover, although neither study showed an overall survival benefit, the Early Breast Cancer Trialists’ Collaborative Group (EBCTCG)^10^ has since reported on a pooled analysis of recent RNI studies, including nearly 11 000 patients, showing reduced overall mortality among those receiving RNI (rate ratio = 0.86; *P* = .002). Together, these findings have been interpreted to support the hypothesis that the benefit of RNI is largely attributable to eradication of occult micrometastatic nodal disease prior to metastatic seeding. The systemic effects of radiation therapy, such as immunologic potentiation,^16^ also remain a possible explanation for these findings.

In considering why RNI, which targets the regional lymph nodes, might appear to exert a more robust effect on DM than on regional recurrences, we look to a broad literature that hints at the potential for underreporting of isolated regional recurrences. Indeed, following primary treatment, there is typically no routine surveillance for nodal recurrences as there is for local recurrences, whereas distant recurrences typically prompt clinical concern only upon development of the signs and symptoms of systemic disease (eg, bone pain, laboratory abnormalities, or gross tumor). For example, internal mammary or upper axillary disease could go undetected by mammography or physical examination for a prolonged period following lumpectomy or mastectomy. In the interim, systemic progression may silently occur, and the window for detecting an isolated regional recurrence missed. Thus, it is possible that regional progression remains occult until symptoms of distant disease prompt evaluation, whereupon the 2 (regional and distant disease) are detected concurrently and are classified only as DM at presentation. Indeed, regional recurrences in landmark breast cancer trials are exceedingly rare: at 5-year follow-up, nodal recurrence was observed in only 1 patient of 891 enrolled in ACOSOG Z0011^6^ and in only 4 among 744 patients enrolled in AMAROS,^5^ all in the context of deescalating axillary management, with concomitantly higher frequencies of local, distant, and survival events.

Our study used PET-CT for nodal evaluation because it is a reproducible noninvasive imaging modality that is performed for most patients with metastatic breast cancer; moreover, recent studies^17,18^ show that it has high diagnostic accuracy in investigating lymph node metastases. Although PET-CT has high specificity for the diagnosis of axillary lymph node involvement, it has low sensitivity for detecting low-volume metastatic disease.^19^ To increase the sensitivity of our imaging analysis, we also evaluated lymph node morphological features on CT images, including cortical thickening and absence of a nodal hilum, which are known to be associated with axillary metastasis.^20^ The combination of functional and anatomical data has been shown to significantly improve the detection of axillary metastases.^21^ Although CT can be informative with regard to axillary disease burden, it is less sensitive than biopsy, such that our findings may ultimately be underestimates of nodal involvement.^22^ On the other hand, because PET-CT may also yield false-positives due to reactive lymphadenopathy,^23^ we characterized a node as positive only if it had the combination of highly suspicious morphologic and metabolic features as described.

### Limitations

Potential drawbacks of this study include the intrinsic limitations of retrospective analyses. Approximately half of all the patients with de novo metastatic breast cancer had evaluable pretreatment PET-CT images and those without such images were excluded, potentially introducing selection bias into the subsequent analyses. That said, all contemporary patients who presented to our center at initial evaluation underwent a PET-CT per national guidelines. In line with the standard of care for metastatic presentations, pathologic evaluation of the axillary lymph nodes was available for only a minority of patients. In addition, given the limited sensitivity of PET-CT for nodal evaluation, it is probable that our report underestimates the proportion of patients with true nodal involvement or, given the limited specificity, potentially misclassifies some with reactive nodes. Of note, despite our hypothesis that nodal disease contributes to distant dissemination, evidence is emerging to suggest that DM can reseed both the primary tumor bed and regional nodes in retrograde fashion.^24^ Although our findings do not preclude the possibility that metastatic disease precedes regional nodal involvement, or that regional and metastatic deposits might arise simultaneously, neither of these alternatives would necessarily exhibit the reduction in DM observed with RNI. Tellingly, the DBCG-IMN trial^13^ evaluated patients who all received axillary and supraclavicular radiation, with or without internal mammary nodal coverage. At 8-year follow-up, overall survival was significantly improved by including the internal mammary nodes (8-year overall survival = 75.9% with internal mammary radiation and 72.2% without; *P* = .005), and the effect was more pronounced in those at higher risk of internal mammary node involvement (despite an absence of clinical involvement at the time of treatment), further suggesting that RT might be acting on occult disease in this basin.^13^

## Conclusions

These findings suggest that most patients with untreated breast cancer who present with DM also exhibit concurrent regional lymph node involvement. This finding, which to our knowledge has not been reported before, does not preclude alternative explanations, but does support the hypothesis that regional nodal disease may precede and contribute to the seeding of distant foci. Adjuvant regional nodal irradiation may, therefore, act to eradicate occult nodal disease and thereby mitigate subsequent distant dissemination. Conversely, the observed modest effect of RNI on regional recurrences is possibly a result of the inherent challenges in surveilling the regional nodal basins, such that nodal recurrences may not be clinically detected until advanced symptoms prompt evaluation, at which point distant dissemination may have already occurred. Further analyses of recurrence patterns among patients receiving various forms of radiotherapeutic or surgical nodal treatment will help to elucidate how regional therapies might be mechanistically linked with distant outcomes.

## Notes

Supplement. **eFigure 1.** Examples of Lymph Node Assessment on CT and PET-CT Images **eFigure 2.** Example of Positive Lymph Nodes Location in PET-CT
