# Supplementary material for: Regional Lymph Node Involvement Among Patients With De Novo Metastatic Breast Cancer
Source: JAMA Netw Open. 2020 Oct 9;3(10):e2018790. doi: 10.1001/jamanetworkopen.2020.18790 (PMC7547365; doi:10.1001/jamanetworkopen.2020.18790)
Supplement: Supplement. — eFigure 1. Examples of Lymph Node Assessment on CT and PET-CT Images eFigure 2. Example of Positive Lymph Nodes Location in PET-CT [file jamanetwopen-e2018790-s001.pdf]

Supplemental Online Content

Bitencourt A, Rossi Saccarelli C, Morris EA, et al. Regional lymph node involvement among patients with de novo metastatic breast cancer. *JAMA Netw Open*. 2020;3(10):e2018790. doi:10.1001/jamanetworkopen.2020.18790

eFigure 1. Examples of Lymph Node Assessment on CT and PET-CT Images
eFigure 2. Example of Positive Lymph Nodes Location in PET-CT

This supplemental material has been provided by the authors to give readers additional information about their work.

© 2020 Bitencourt A et al. *JAMA Network Open.*

SUPPLEMENTARY FIGURES

eFigure 1. Examples of Lymph Node Assessment on CT and PET-CT Images

A-B) unlikely; C–D) less likely; E–F) possible; G–H) suspicious; I–J) consistent with.

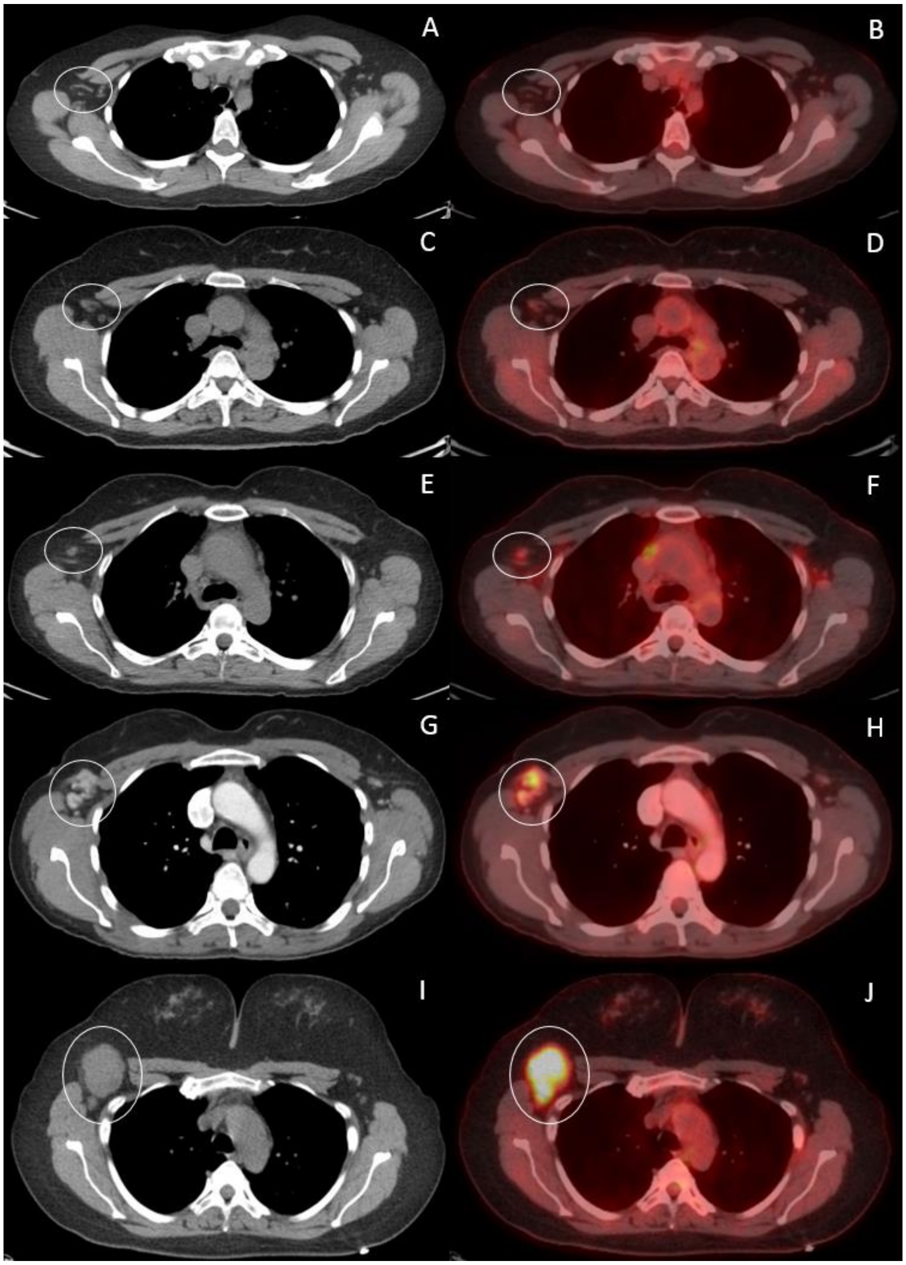

© 2020 Bitencourt A et al. *JAMA Network Open.*

eFigure 2. Example of Positive Lymph Nodes Location in PET-CT

A) Level I axillary enlarged lymph nodes (arrow); B) Right internal mammary chain (arrow); C) Level II axillary lymph nodes (arrow); D) Level III axillary lymph node (arrow); E) Right supraclavicular lymph nodes (arrows).

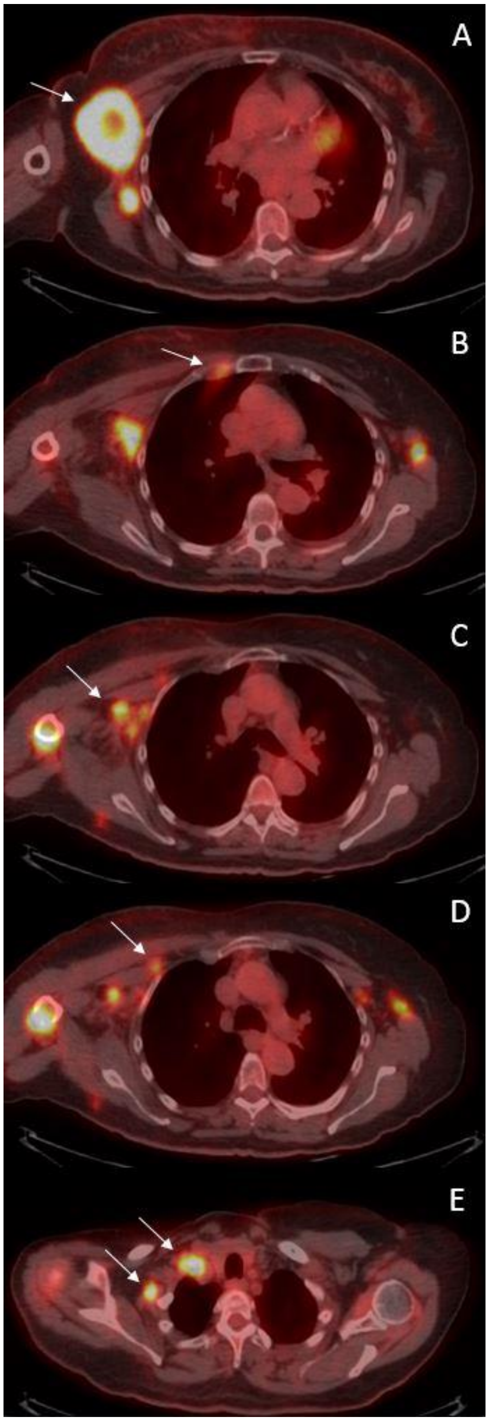

© 2020 Bitencourt A et al. *JAMA Network Open.*
